# Neurohistopathological Alterations Induced by Clitoria Ternatea and Emblica Officinalis Extracts in Diabetic Male Wistar Rats

**DOI:** 10.7759/cureus.72079

**Published:** 2024-10-21

**Authors:** Ravi Kiran, Vishali Neelakandan, Bandarupalli Naveen Kumar, Edward Indla

**Affiliations:** 1 Department of Anatomy, Meenakshi Academy of Higher Education and Research, Chennai, IND; 2 Department of Anatomy, Vels Medical College, Chennai, IND; 3 Department of Anatomy, Mamata Academy of Medical Sciences, Hyderabad, IND; 4 Department of Anatomy, Mamata Medical College, Khammam, IND

**Keywords:** diabetes, gliosis, neuronal degeneration, neuroprotection, traditional medicine, vacuolation

## Abstract

Background

Diabetes mellitus is a chronic condition that can lead to significant neurodegenerative complications. The extracts of traditional medicinal plants such as *Clitoria ternatea* and *Emblica officinalis* have neuroprotective properties. This study investigates the neurohistopathological alterations in diabetic male Wistar rats treated with these extracts.

Objective

This study aimed to assess the neuroprotective potential of *Clitoria ternatea* and *Emblica officinalis* extracts in mitigating neurohistopathological alterations in streptozotocin-induced diabetic male Wistar rats

Methods

In this experimental investigation, 48 male Wistar rats, aged 8-12 weeks and weighing 200-250g, were randomly allocated into eight groups (n=6): control, diabetic model, metformin treatment, *Clitoria ternatea* extract treatment, *Emblica officinalis* extract treatment, diabetic model + *Clitoria ternatea *extract treatment, diabetic model + *Emblica officinalis *extract treatment, and diabetic model + combined extract treatment. Streptozotocin 40 mg/kg was used to induce diabetes. Treatments included 100 mg/kg metformin, 300 mg/kg *Clitoria ternatea* extract, and 250 mg/kg *Emblica officinalis* extract. After three weeks, blood glucose levels, body weight, and neurohistopathological parameters (neuronal degeneration, gliosis, and vacuolation) were evaluated.

Results

The diabetic rats had much higher blood sugar levels (320.4 ± 15.3 mg/dL) and lost a lot of weight (180.7 ± 9.5 g) compared to the control group, which had normal blood sugar levels (92.3 ± 4.8 mg/dL) and stable body weight (225.4 ± 12.1 g) (p < 0.001). Treatment with *Clitoria ternatea* extract reduced blood glucose to 160.3 ± 12.1 mg/dL, while *Emblica officinalis* extract lowered it to 155.4 ± 11.8 mg/dL. The combined treatment brought glucose levels closer to normal (110.5 ± 8.9 mg/dL). Similarly, both extracts mitigated weight loss, with the combined treatment maintaining body weight at 220.4 ± 12.0 g, close to the control group (p < 0.05). The study of diabetic rats that had not been treated showed severe neuronal degeneration (4.2±0.4), gliosis (4.5±0.5), and vacuolation (4.7±0.5). Treatment groups showed marked reductions in these parameters, with the combined treatment producing the greatest neuroprotective effects, comparable to controls (p < 0.001).

Conclusions

Extracts from *Clitoria ternatea* and *Emblica officinalis*, particularly when combined, provide significant neuroprotective advantages in male Wistar diabetic rats, significantly lowering hyperglycemia and related neurohistopathological changes. These findings support the potential use of these extracts as complementary or alternative therapies in managing diabetic complications.

## Introduction

Diabetes mellitus (DM) is a type of chronic metabolic disease characterized by persistent hyperglycemia caused either due to the insufficiency or resistance factor function of insulin. It is a major global health problem that affects millions around the world and results in serious sequelae, such as neuropathy, retinopathy, nephropathy, and cardiovascular diseases [1]. Diabetic neuropathy, which is one of the most disabling features, as being related to neurohistopathological abnormalities may bring about considerable burden on a baby's entire brain [2]. Most of these neurodegenerative changes mainly result from oxidative stress, inflammation, and the accumulation of advanced glycation end-products (AGEs) which cause neuronal injury [3].

The pathogenesis of diabetic neuropathy is complex and multifactorial. In addition to oxidative stress and inflammation, metabolic dysfunction has been linked to neurodegenerative disorders, such as Alzheimer's disease, which shares pathways with diabetes-related neurodegeneration [4]. This complexity underscores the need for effective therapies to manage diabetic complications. However, the effectiveness of current pharmaceutical treatments for diabetes and its complications is frequently insufficient, and they often have unfavorable side effects [5]. As a result, interest in alternative and complementary therapies has increased.

*Clitoria ternatea* (butterfly pea) and *Emblica officinalis* (Indian gooseberry or amla) are two medicinal plants extensively used in traditional Ayurvedic medicine that have shown promise in preclinical studies. *Clitoria ternatea* is renowned for its antioxidant, anti-inflammatory, and neuroprotective effects [6], suggesting it may play a role in addressing oxidative stress and inflammation, key factors in the development of diabetic neuropathy. Similarly, *Emblica officinalis* is well-known for its rich vitamin C content and strong antioxidant properties, which have been shown to mitigate oxidative stress associated with diabetic complications like neuropathy, retinopathy, and cardiovascular diseases [6, 7]. The antioxidant properties of *Emblica officinalis* help counteract the excessive production of reactive oxygen species (ROS) that result from prolonged hyperglycemia. By scavenging these free radicals, *Emblica officinalis* reduces oxidative damage to cells, including neurons, which are particularly vulnerable in diabetic conditions. Studies have demonstrated that *Emblica officinalis* can lower markers of oxidative stress, improve glucose metabolism, and protect tissues from damage, making it a promising natural intervention for mitigating the complications associated with diabetes. Its rich antioxidant profile not only preserves cellular function but also supports the body's natural defenses against the inflammatory responses that often accompany diabetic complications [7]. These effects are critical in preventing neuronal degeneration, a hallmark of diabetic neuropathy.

While the general health benefits of *Clitoria ternatea* and *Emblica officinalis* and their effects on diabetes have been studied, their specific neuroprotective potential in diabetic neuropathy remains underexplored. Previous studies have emphasized a heightened risk of neurodegenerative disorders, including Alzheimer’s, in individuals with type 2 diabetes [8], emphasizing the importance of investigating neuroprotective interventions for diabetic patients. Additionally, the role of neuroinflammation in memory disruption and cognitive decline in diabetic and aging populations supports the need for anti-inflammatory and neuroprotective therapies [9].

This study aims to evaluate the potential neuroprotective effects of *Clitoria ternatea* and *Emblica officinalis* extracts in diabetic neuropathy. Specifically, the study investigates the neurohistopathological alterations in streptozotocin-induced diabetic male Wistar rats after treatment with these extracts. The objectives include assessing the effects of *Clitoria ternatea* and *Emblica officinalis *extracts on blood glucose levels, evaluating their ability to reduce neuronal degeneration, gliosis, and vacuolation in the brain, and examining the combined efficacy of these extracts in mitigating the neurohistopathological changes associated with diabetic neuropathy.

## Materials and methods

This experiment aimed to explore the neurohistopathological changes in the brains of male Wistar rats induced with diabetes using streptozotocin (STZ). STZ-induced diabetic rats are commonly used to mimic Type 1 diabetes (T1D) in experimental research. STZ is a chemical compound that selectively targets and destroys insulin-producing beta cells in the pancreas, leading to reduced or absent insulin production, which mimics the pathophysiological features of T1D. In T1D, the body’s immune system attacks and destroys the beta cells of the pancreas, resulting in a lack of insulin. Similarly, STZ causes beta cell death, leading to hyperglycemia and other metabolic alterations, allowing researchers to study T1D's effects and potential treatments in animal models. Thus, while STZ-induced diabetes is a useful model for studying aspects of T1D, especially insulin deficiency, it does not entirely reflect the autoimmune mechanisms underlying the disease.

After inducing diabetes, the rats were treated with extracts from *Clitoria ternatea* and *Emblica officinalis* to assess their neuroprotective effects. The study was conducted at Vyas Labs in Hyderabad, Telangana (India), between March 12, 2023, and June 17, 2023. A randomized controlled trial (RCT) design was employed to ensure rigorous scientific methodology and to enhance the internal validity of the findings. Randomization was used in this study to reduce bias and make sure that the effects seen on neuropathological parameters were due to the treatments and not to outside factors. This design strengthened the reliability and robustness of the conclusions regarding the potential therapeutic benefits of *Clitoria ternatea* and *Emblica officinalis* in mitigating diabetic neuropathy.

Assessment of LD50

The acute toxicity of *Clitoria ternatea* and *Emblica officinalis* extracts was tested in albino mice of both sexes (16-20 g) maintained under standard husbandry conditions. Mice were fasted for 3 hours prior to the experiment and extracts were administered in a single dose. The mice were observed for 48 hours to determine whether any acute toxicological effects were present. According to these observations, the further dosing was adjusted according to OECD Guideline No. 420 to a limit dose of 2000 mg/kg [10].

From the lethal dose 50 (LD50) determination, the selected doses for the study were 300 mg/kg for *Clitoria ternatea* and 250 mg/kg for *Emblica officinalis*. These doses were chosen based on preliminary trials and found to be effective in producing significant biological responses without causing adverse effects.

Animal models and grouping

We selected male Wistar rats, 8-12 weeks of age and weighing between 200-250 grams, for this study. Rats were housed in an environment with 12 hours of light per day, temperature was maintained at 22 ± 2°C, and relative humidity set around a range of between 50%-55%. Animals were allowed food and water ad libitum.

Based on the nature of the study and the expected effect sizes observed in preliminary experiments, we conducted a power analysis to determine the appropriate sample size. Using an estimated effect size of 1.5 (Cohen’s d) for the primary outcomes (blood glucose levels and histopathological changes), a power of 0.8, and an alpha value of 0.05, the power calculation indicated that six animals per group would be sufficient to detect statistically significant differences.

A total of 48 male rats were randomly assigned into eight groups (n = 6 per group). Group I (control group) had a control diet to which no stress was applied. Group II (diabetic model) received Streptozotocin at 40 mg/kg. Group III was treated with Metformin 100 mg/kg. Group IV received 300 mg/kg of the* Clitoria ternatea* extract. Group V was treated with 250 mg/kg of the *Emblica officinalis*. Group VI (diabetic model + *Clitoria ternatea* extract) was administered 40 mg/kg streptozotocin with 300 mg/kg of the *Clitoria ternatea* extract. Group VII (diabetic model + *Emblica officinalis* extract) was administered 40 mg/kg streptozotocin with 250 mg/kg of the *Emblica officinalis* extract. Group VIII (diabetic model + combined extract treatment) was administered 40 mg/kg streptozotocin with 300 mg/kg of the *Clitoria ternatea* and 250 mg/kg of the *Emblica officinalis* extracts (Table 1). 

**Table 1:** Overview of experimental groups, descriptions, number of rats, treatment types, and dosages

Group	Description	Number of Rats	Treatment	Dosage
I	Control	6	Standard diet	None
II	Diabetic Model	6	streptozotocin administration	40 mg/kg of streptozotocin
III	Metformin Treatment	6	Metformin	100 mg/kg of metformin
IV	*Clitoria ternatea* Extract Treatment	6	*Clitoria ternatea* extract	300 mg/kg of the extract
V	*Emblica officinalis* Extract Treatment	6	*Emblica officinalis* extract	250 mg/kg of the extract
VI	Diabetic Model + *Clitoria ternatea* Extract	6	streptozotocin + *Clitoria ternatea* extract	40 mg/kg streptozotocin + 300 mg/kg *Clitoria ternatea* extract
VII	Diabetic Model + *Emblica officinalis* Extract	6	streptozotocin + *Emblica officinalis* extract	40 mg/kg streptozotocin + 250 mg/kg *Emblica officinalis* extract
VIII	Diabetic Model + Combined Extract Treatment	6	streptozotocin + *Clitoria ternatea* + *Emblica officinalis* extracts	40 mg/kg streptozotocin + 300 mg/kg *Clitoria ternatea* + 250 mg/kg *Emblica officinalis* extracts

Groups II, III, VI, VII, and VIII were made diabetic by intraperitoneal injections of streptozotocin at a dosage of 40 mg/kg body weight. Blood glucose levels were measured 72 hours post-injection to confirm diabetic induction. Metformin was orally administered to Group III rats at a dosage of 100 mg/kg. *Clitoria ternatea* and *Emblica officinalis* extracts were solubilized in water and orally administered at concentrations of 300 mg/kg and 250 mg/kg, respectively. Blood glucose levels and body weight were monitored at baseline, 14 days, and 21 days. At the end of the 21-day experiment, animals were euthanized, and their brains were harvested. The extracted brain tissues were stained using cresyl violet and examined under a microscope for histopathological changes, including assessments of neuronal degeneration, gliosis, and vacuolation.

This study received approval from the Institute Animal Ethical Committee (IAEC/VL/7/2022-23) and was conducted at Vyas Labs, Hyderabad, Telangana, India,

Statistical analysis was performed using one-way ANOVA to assess differences among the treatment groups for blood glucose levels, body weight, and histopathological parameters. Tukey's post hoc test was applied to identify significant differences between groups, and a p-value of <0.05 was considered statistically significant.

## Results

Significantly higher blood glucose levels in Groups II, VI, VII, and VIII compared to the control group confirmed the induction of diabetes (p=0.001). Post-treatment evaluations on days 14 and 21 demonstrated significant reductions in blood glucose levels in the treatment groups. Group III (metformin), Group IV (*Clitoria ternatea* extract), Group V (*Emblica officinalis* extract), and Group VIII (combined extracts) all exhibited notable decreases in blood glucose levels compared to the diabetic model group (Group II) (p < 0.05). The biggest drop was seen in Group VIII (combined extracts). By day 21, their blood sugar levels were almost the same as the control group, which means they got better (p < 0.001) (Table 2).

**Table 2:** Comparison of initial and final blood glucose levels (mg/dL) across different treatment groups Diabetic rats treated with extracts of *Clitoria ternatea*, *Emblica officinalis*, or their combination showed significant reductions in blood glucose levels, with the combined extract treatment group achieving near-normal levels. p-values: Significant difference compared to the diabetic model (Group II) for all treatment groups (p < 0.05). Highly significant reduction in Group VIII (p < 0.001).

Group	Description	Initial Blood Glucose (mg/dL)	Final Blood Glucose (mg/dL)
I	Control	90.5 ± 5.2	92.3 ± 4.8
II	Diabetic Model	92.1 ± 6.0	320.4 ± 15.3
III	Metformin Treatment	90.8 ± 5.5	140.7 ± 10.2
IV	*Clitoria ternatea* Extract Treatment	91.3 ± 4.9	160.3 ± 12.1
V	*Emblica officinalis* Extract Treatment	89.7 ± 5.7	155.4 ± 11.8
VI	Diabetic Model + *Clitoria ternatea*	90.2 ± 5.3	130.8 ± 9.7
VII	Diabetic Model + *Emblica officinalis*	90.5 ± 5.4	135.2 ± 10.1
VIII	Diabetic Model + Combined Extracts	91.0 ± 5.6	110.5 ± 8.9

Diabetic rats (Groups II, VI, VII, and VIII) exhibited significant weight loss compared to the control group (p < 0.001). Metformin (Group III), *Clitoria ternatea* (Group IV), and *Emblica officinalis* (Group V) treatments slowed down this weight loss, and by the 14th day, there were clear differences. The combined extract treatment (Group VIII) did a great job of keeping body weight close to that of the control group. By the 21st day, there was a highly significant retention of body weight (p < 0.001) (Table 3).

**Table 3:** Initial and final body weights (g) of different treatment groups Diabetic rats exhibited significant weight loss, while treatment with *Clitoria ternatea*, *Emblica officinalis*, and their combination effectively mitigated this loss, with the combined extract group showing weight levels close to those of the control group. p-values: Significant improvement in body weight compared to the diabetic model (Group II) for all treatment groups (p < 0.05). Highly significant improvement in Group VIII (p < 0.001).

Group	Description	Initial Body Weight (g)	Final Body Weight (g)
I	Control	215.6 ± 10.3	225.4 ± 12.1
II	Diabetic Model	217.8 ± 11.0	180.7 ± 9.5
III	Metformin Treatment	216.4 ± 10.8	210.3 ± 11.6
IV	*Clitoria ternatea* Extract Treatment	214.7 ± 10.1	205.8 ± 10.4
V	*Emblica officinalis* Extract Treatment	215.5 ± 10.6	207.3 ± 10.9
VI	Diabetic Model + *Clitoria ternatea*	216.1 ± 10.4	212.9 ± 11.2
VII	Diabetic Model + *Emblica officinalis*	217.3 ± 10.7	211.6 ± 11.5
VIII	Diabetic Model + Combined Extracts	215.8 ± 10.9	220.4 ± 12.0

**Figure 1:**
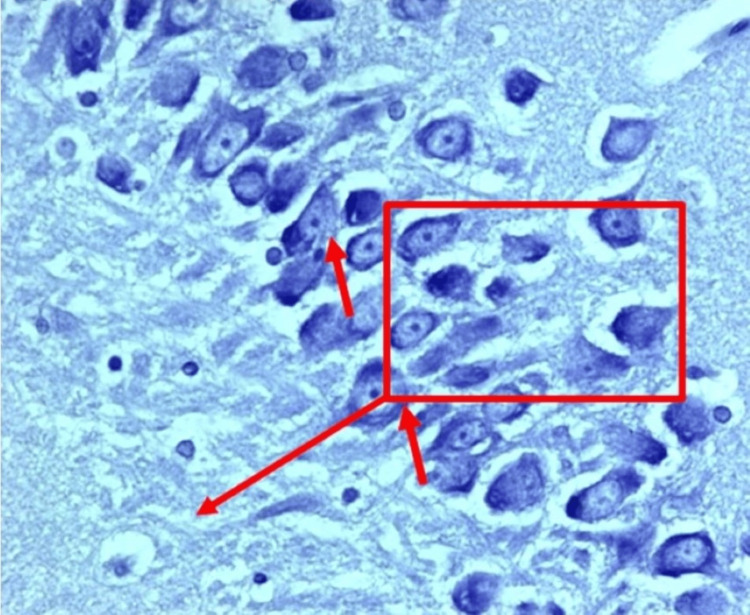
Typical morphological features of pyramidal neurons in the rat hippocampus, control group

Histopathological examination of brain tissues revealed severe neurodegenerative changes in the diabetic rats (Group II) compared to the control group (Group I). These included marked neuronal degeneration, gliosis, and vacuolation. Neuroprotective effects were seen in treatments with *Clitoria ternatea* (Group VI), *Emblica officinalis* (Group VII), and the combined extracts (Group VIII). On the 14th day, there was some neuroprotection, and by the 21st day, there were huge improvements in Group VIII, whose histological structure was very similar to that of the control group (p < 0.001) (Figure 1).

**Figure 2:**
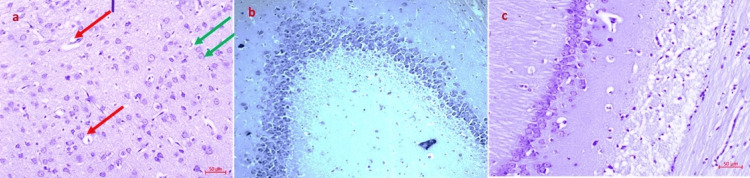
Histopathological analysis of brains of rats from Group II, III, and VI a) Multiple dying neurons in the dentate gyrus and CA3 area of the hippocampus in Wistar rats given streptozotocin; b) changes in the shape of pyramidal neurons in all hippocampal areas of Wistar rats given metformin; c) a moderate rise in pyramidal neuron proliferation in the CA4 area of the hippocampus after Clitoria ternatea treatment in streptozotocin-induced diabetic Wistar rats

The neurons in Group II were severely damaged, as shown by the fact that they were getting smaller and had pyknotic nuclei and a vacuolated cytoplasm. By the 14th day, treatment with metformin (Group III), *Clitoria ternatea* (Group IV), and *Emblica officinalis* (Group V) extracts had greatly slowed down the loss of neurons (Figure 2a-c). 

On the 21st day, the combined treatment (Group VIII) had the least neuronal degeneration, which was significantly different from the diabetic model group (p < 0.001) (Table 4).

**Table 4:** Neuronal degeneration scores across different treatment groups Diabetic rats showed severe neuronal degeneration, while treatment with *Clitoria ternatea*, *Emblica officinalis*, and their combination significantly reduced degeneration, with the combined extract group achieving scores closest to the control group. p-values: Significant reduction in neuronal degeneration compared to the diabetic model (Group II) for all treatment groups (p < 0.05). Highly significant reduction in Group VIII (p < 0.001).

Group	Description	Neuronal Degeneration Score
I	Control	0.5 ± 0.1
II	Diabetic Model	4.2 ± 0.4
III	Metformin Treatment	1.8 ± 0.3
IV	*Clitoria ternatea* Extract Treatment	2.0 ± 0.3
V	*Emblica officinalis* Extract Treatment	1.9 ± 0.3
VI	Diabetic Model + *Clitoria ternatea*	1.2 ± 0.2
VII	Diabetic Model + *Emblica officinalis*	1.3 ± 0.2
VIII	Diabetic Model + Combined Extracts	0.8 ± 0.1

**Figure 3:**
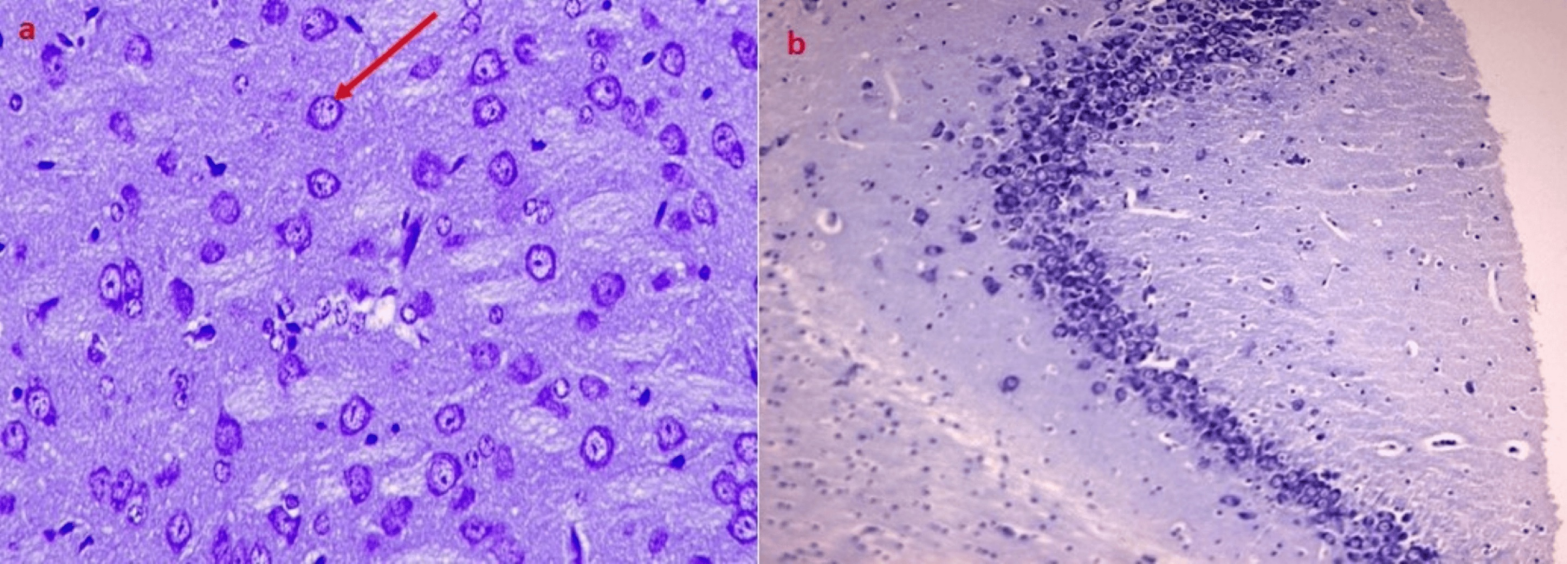
Histopathological analysis of brains of rats from Group IV and VII a) Moderate proliferation of pyramidal neurons in the CA4 area of the hippocampus due to Emblica officinalis administration in streptozotocin-induced diabetic Wistar rats; b) normal morphological characteristics of pyramidal neurons throughout the hippocampus in rats treated with Clitoria ternatea

In the diabetic model group (Group II), there was clear gliosis, which was shown by more glial cells (Figure 3).

Metformin (Group III), *Clitoria ternatea* (Group IV), and *Emblica officinalis* (Group V) treatments all significantly decreased gliosis by the 14th day. The combined extracts treatment (Group VIII) showed the most significant reduction by the 21st day, almost reaching levels seen in the control group (p < 0.001) (Table 5).

**Table 5:** Gliosis scores in different treatment groups Diabetic rats demonstrated a significant increase in gliosis, whereas treatment with *Clitoria ternatea*, *Emblica officinalis*, and their combination effectively reduced gliosis, with the combined extract group showing the most pronounced reduction, nearing control group levels. p-values: Significant reduction in gliosis compared to the diabetic model (Group II) for all treatment groups (p < 0.05). Highly significant reduction in Group VIII (p < 0.001).

Group	Description	Gliosis Score
I	Control	0.4 ± 0.1
II	Diabetic Model	4.5 ± 0.5
III	Metformin Treatment	2.0 ± 0.4
IV	*Clitoria ternatea* Extract Treatment	2.2 ± 0.4
V	*Emblica officinalis* Extract Treatment	2.1 ± 0.3
VI	Diabetic Model + *Clitoria ternatea*	1.5 ± 0.3
VII	Diabetic Model + *Emblica officinalis*	1.4 ± 0.3
VIII	Diabetic Model + Combined Extracts	0.9 ± 0.2

**Figure 4:**
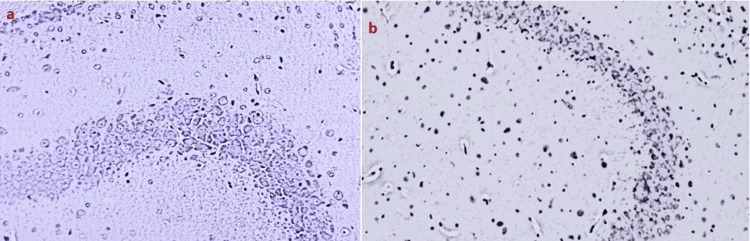
Histopathological analysis of brains of rats from Group VII and VIII a) Intense hyperplasia and proliferation of pyramidal neurons in the CA3 region of the hippocampus following Emblica officinalis treatment in diabetic rats; b) significant proliferation of pyramidal neurons in the hippocampus of diabetic rats treated with combined extracts of Clitoria ternatea and Emblica officinalis

Extensive vacuolation, indicative of cellular edema and necrosis, was observed in Group II. Treatment with metformin (Group III), *Clitoria ternatea* (Group IV), and *Emblica officinalis* (Group V) significantly ameliorated vacuolation by the 14th day (Figure 4).

The combined extracts treatment (Group VIII) showed the most substantial reduction in vacuolation by the 21st day, with histological features resembling the control group (p < 0.001) (Table 6).

**Table 6:** Vacuolation scores for the various treatment groups Diabetic rats displayed marked vacuolation, signifying cell damage. Treatments with *Clitoria ternatea*, *Emblica officinalis*, and their combination resulted in a significant decrease in vacuolation, with the combined treatment showing the closest results to the control group. p-values: Significant reduction in vacuolation compared to the diabetic model (Group II) for all treatment groups (p < 0.05). Highly significant reduction in Group VIII (p < 0.001).

Group	Description	Vacuolation Score
I	Control	0.3 ± 0.1
II	Diabetic Model	4.7 ± 0.5
III	Metformin Treatment	1.9 ± 0.3
IV	*Clitoria ternatea* Extract Treatment	2.1 ± 0.4
V	*Emblica officinalis* Extract Treatment	2.0 ± 0.3
VI	Diabetic Model + *Clitoria ternatea*	1.4 ± 0.3
VII	Diabetic Model + *Emblica officinalis*	1.3 ± 0.3
VIII	Diabetic Model + Combined Extracts	0.8 ± 0.2

The statistical analysis confirmed the significance of the observed histopathological changes. The one-way ANOVA results indicated significant differences among the groups for blood glucose levels, body weight, and histopathological parameters (p < 0.05). Tukey's post hoc test further highlighted the highly significant efficacy of the combined extracts treatment (Group VIII) over individual treatments and the diabetic model group, particularly on the 14th and 21st days.

## Discussion

The study demonstrates that *Clitoria ternatea* and *Emblica officinalis* extracts protect neurons in male Wistar rats that have been given streptozotocin to make them diabetic. STZ-induced diabetic rats are widely used as an experimental model to simulate T1D. STZ specifically targets and destroys the insulin-producing beta cells in the pancreas, leading to reduced or absent insulin production, thereby mimicking the key features of T1D. In T1D, the body's immune system attacks and destroys the beta cells of the pancreas, resulting in an insulin deficiency. Similarly, STZ induces beta cell death, causing hyperglycemia and other metabolic disturbances, making it a valuable model for studying T1D and testing potential treatments.

Both extracts effectively reduced hyperglycemia, mitigated weight loss, and protected against neurohistopathological alterations, such as neuronal degeneration, gliosis, and vacuolation. The most pronounced effects were observed when the extracts were used in combination, indicating a potential synergistic relationship [11]. While metformin is traditionally associated with the treatment of T2D, recent studies have explored its beneficial effects in T1D models, particularly with improving insulin sensitivity, reducing hepatic glucose production, and exerting anti-inflammatory and antioxidant effects. Given these emerging findings, we chose metformin as a comparative treatment to evaluate its potential neuroprotective effects alongside *Clitoria ternatea* and *Emblica officinalis* extracts [11-13].

The extracts, both individually and in combination, significantly lowered blood glucose levels in diabetic rats. This result aligns with the previously documented hypoglycemic properties of these plants. *Clitoria ternatea* has been shown to enhance insulin secretion and sensitivity, while *Emblica officinalis* is rich in antioxidants, particularly vitamin C, which plays a key role in glucose metabolism [12]. The group receiving the combined treatment of *Clitoria ternatea* and *Emblica officinalis* exhibited blood glucose levels that were nearly equivalent to those of the control group, indicating that these extracts have the potential to serve as a viable alternative to conventional antidiabetic medications like metformin. By the 21st day of treatment, the reduction in blood glucose levels was highly significant, further demonstrating the effectiveness of the extracts in managing hyperglycemia. These findings suggest that the combined use of these natural extracts could play a crucial role in regulating blood sugar levels and offer a promising therapeutic option for diabetes management [13].

The weight loss typically associated with diabetes, often due to the body's inability to properly utilize glucose, was significantly reduced in the rats treated with *Clitoria ternatea* and *Emblica officinalis* extracts. This reduction in weight loss suggests an improvement in metabolic function, likely driven by enhanced glucose utilization and a decrease in muscle breakdown (catabolism). Notably, the rats in the combined treatment group maintained body weight levels that were nearly identical to those of the healthy control group, highlighting the strong therapeutic potential of these extracts. These findings not only reinforce the role of *Clitoria ternatea* and *Emblica officinalis* as effective antidiabetic agents but also suggest that they may contribute to improved overall health and nutritional status by preventing the adverse effects of diabetes-related weight loss [14].

The study found a significant reduction in neuronal degeneration in the treated groups, with the combined treatment producing the most substantial effect. The antioxidant and anti-inflammatory properties of both *Clitoria ternatea* and *Emblica officinalis* [15] stopped neuronal damage caused by diabetes, which is often caused by oxidative stress and inflammation. The decrease in neuronal damage, especially in the combined group, suggests strong neuroprotection that was most likely fueled by synergistic effects [16]. Gliosis, characterized by the proliferation of glial cells in response to neuronal injury, was notably reduced in the treated rats. This reduction is significant because gliosis can exacerbate neuroinflammation and contribute to further neuronal damage. The lower amount of gliosis in the groups that were treated with *Clitoria ternatea* and *Emblica officinalis* shows that the plants have a strong neuroprotective effect, which could have therapeutic implications for diabetic neuropathy [17].

Cellular vacuolation, which is indicative of cellular damage, was significantly reduced in the treated rats. The near-elimination of vacuolation in the combined treatment group further emphasizes the protective effects of these extracts. This suggests a potent protective mechanism that could have therapeutic value, particularly when the extracts are used together [18]. The highly significant reduction in vacuolation supports the possibility of synergistic interactions between the extracts, warranting further investigation [19].

Although the exact mechanisms behind the neuroprotective effects of *Clitoria ternatea* and *Emblica officinalis* are not fully understood, their rich phytochemical composition, which includes flavonoids, tannins, and vitamin C, likely plays a key role. These compounds are known to reduce oxidative stress, modulate inflammation, and enhance neurotrophic factors that support neuronal survival and function [20]. The synergistic effects observed in the combined treatment group suggest that these phytochemicals may interact in ways that amplify their neuroprotective properties [21].

The short study duration restricts insights into the long-term effects of the treatments. Moreover, the precise molecular mechanisms responsible for the observed neuroprotective effects were not explored and warrant further investigation. The small sample size also limits the generalizability of the findings, highlighting the need for larger-scale studies to validate these results.

## Conclusions

The findings of this study suggest that *Clitoria ternatea* and *Emblica officinalis* extracts, particularly when combined, offer significant neuroprotective benefits in diabetic male Wistar rats. These extracts effectively reduce hyperglycemia, prevent weight loss, and mitigate neurohistopathological alterations associated with diabetic neuropathy. The highly significant results observed with the combined treatment highlight the potential of these traditional medicinal plants as complementary or alternative therapies for managing diabetes and its complications.
